# Detection of IFNγ-Secreting CD4^+^ and CD8^+^ Memory T Cells in COVID-19 Convalescents after Stimulation of Peripheral Blood Mononuclear Cells with Live SARS-CoV-2

**DOI:** 10.3390/v13081490

**Published:** 2021-07-29

**Authors:** Victoria Matyushenko, Irina Isakova-Sivak, Igor Kudryavtsev, Arina Goshina, Anna Chistyakova, Ekaterina Stepanova, Polina Prokopenko, Ivan Sychev, Larisa Rudenko

**Affiliations:** Department of Virology, Institute of Experimental Medicine, 197376 Saint Petersburg, Russia; matyshenko@iemspb.ru (V.M.); igorek1981@yandex.ru (I.K.); arina8goshina@gmail.com (A.G.); anna.k.chistiakova@gmail.com (A.C.); fedorova.iem@gmail.com (E.S.); pi.prokopenko@gmail.com (P.P.); sychev.ia@iemspb.ru (I.S.); vaccine@mail.ru or

**Keywords:** SARS-CoV-2, PBMC, COVID-19 convalescents, T-cell memory, T-helper long-lived immunity

## Abstract

Background: New coronavirus SARS-CoV-2, a causative agent of the COVID-19 pandemic, has been circulating among humans since November 2019. Multiple studies have assessed the qualitative and quantitative characteristics of virus-specific immunity in COVID-19 convalescents, however, some aspects of the development of memory T-cell responses after natural SARS-CoV-2 infection remain uncovered. Methods: In most of published studies T-cell immunity to the new coronavirus is assessed using peptides corresponding to SARS-CoV-1 or SARS-CoV-2 T-cell epitopes, or with peptide pools covering various parts of the viral proteins. Here, we determined the level of CD4^+^ and CD8^+^ memory T-cell responses in COVID-19 convalescents by stimulating PBMCs collected 1 to 6 months after recovery with sucrose gradient-purified live SARS-CoV-2. IFNγ production by the central and effector memory helper and cytotoxic T cells was assessed by intracellular cytokine staining assay and flow cytometry. Results: Stimulation of PBMCs with live SARS-CoV-2 revealed IFNγ-producing T-helper effector memory cells with CD4^+^CD45RA^−^CCR7^−^ phenotype, which persisted in circulation for up to 6 month after COVID-19. In contrast, SARS-CoV-2-specific IFNγ-secreting cytotoxic effector memory T cells were found at significant levels only shortly after the disease, but rapidly decreased over time. Conclusion: The stimulation of immune cells with live SARS-CoV-2 revealed a rapid decline in the pool of effector memory CD8^+^, but not CD4^+^, T cells after recovery from COVID-19. These data provide additional information on the development and persistence of cellular immune responses after natural infection, and can inform further development of T cell-based SARS-CoV-2 vaccines.

## 1. Introduction

The ongoing COVID-19 pandemic resulted in severe socio-economic crisis around the world. To date, over 190 million cases with more than 4 million fatalities have been registered worldwide, with many countries struggling in their third pandemic waves [1]. Specific SARS-CoV-2 vaccines have been developed with an unprecedented speed, and several safe and effective candidates have been released to the market less than a year since the virus emerged [2,3]. The human immune responses to natural SARS-CoV-2 infection and vaccination with various vaccines have been extensively studied and a huge amount of data accumulated, regarding the magnitude and the duration of antibody immunity, as well as T cell-based responses (reviewed in [4,5]). It should be noted that the vast majority of experiments on T-cell immunity is carried out using either selected T-cell epitopes or peptide pools representing some of the immunogenic loci of SARS-CoV-2 proteome [6,7,8]. However, the use of a live virus antigen for the stimulation of T cells with phenotyping the pool of memory T cells can be another promising tool for assessing cellular immunity after infection or vaccination. Here, we report the preliminary findings on the detection of virus-specific memory T cells after stimulation of peripheral blood mononuclear cells (PBMC) of COVID-19 convalescents with live SARS-CoV-2 isolated in the first COVID-19 wave.

## 2. Materials and Methods

### 2.1. Study Participants and Sample Collection

Thirty COVID-19 convalescents and fifteen naïve blood donors aged 21 to 77 years participated in the current study (Table S1). Among them, seven individuals who had recovered from severe disease, i.e., they were diagnosed with pneumonia with lung infiltrate > 50% on computer tomography. The vast majority of volunteers did not have any chronic diseases prior to the infection. There was one subject with atopic dermatitis, one with chronic bronchitis and one with Hashimoto’s thyroiditis; all diseases were in the stage of no exacerbation or remission. One subject from the naïve group also had a hypertonic disease (Table S1).

Peripheral blood was collected between 1 and 6 months after the COVID-19 onset. PBMCs were isolated by standard procedure using Lymphocytes Separation Media (Capricorn, Germany). PBMCs were resuspended in CR-10 media (RPMI supplemented with 10% FBS, 5 mM HEPES, 1× antibiotic-antimycotic, 50 µM β-mercaptoethanol, and 40 U/mL human IL-2) at a concentration 2 × 10^7^ cells/mL, and 100 µL of this suspension were added to each well of a V-bottom 96-well plate (Sarstedt, Germany) for further intracellular cytokine staining assay (ICS), as described below. Serum samples were collected from the same individuals to assess antibody immune responses to SARS-CoV-2. The study was approved by the local ethical committee of the Institute of Experimental Medicine (protocol #№2/20 dated 7 April 2020), and all participants signed an informed consent.

### 2.2. SARS-CoV-2 Virus Propagation, Purification and Titration

The SARS-CoV-2 isolate hCoV-19/St_Petersburg-3524S/2020 (GISAID EPI_ISL_415710) was obtained from Smorodintsev Research Institute of Influenza (Saint Petersburg, Russia). Virus was propagated on Vero CCL81 cells using DMEM/2%FBS (DMEM supplemented with 1×antibiotic-antimycotic, 10 mM HEPES and 2% FBS, all from Gibco, USA), at a multiplicity of infection (MOI) 0.005. Virus-infected cells were incubated at 37 °C, 5% CO_2_ for 72 h, and then the cell supernatants were collected and clarified by low-speed centrifugation. The virus was pelleted by centrifugation at 19,000 rpm, 4 °C for 4 h using ultracentrifuge (Beckman Coulter, Brea, CA, USA). After the supernatant was discarded, the pellet was resuspended in 1 mL of sterile PBS, followed by virus concentration on a sucrose gradient prepared using HEPES/EDTA buffer (PBS supplemented with 25 mM HEPES and 5 mM EDTA). For this, one part of the resuspended virus was layered on 4 parts of 30% sucrose and 4 parts of 60% sucrose, followed by centrifugation at 35,000 rpm, 4 °C for 20 h. Then, the interphase was collected and additionally washed with PBS by centrifugation at 35,000 rpm, 4 °C for 1 h. The final precipitate containing live SARS-CoV-2 was resuspended in PBS and stored in single-use aliquots at −70 °C.

Virus titer was determined by 50% Tissue Culture Infection Dose (TCID_50_) assay as described elsewhere [9]. Briefly, 10-fold dilutions of SARS-CoV-2 prepared on the DMEM/2%FBS were inoculated into 95%-confluent monolayer of Vero-CCL81 cells seeded on a 96-well cell-culture plate, followed by incubation at the 37 °C and 5%CO_2_ for 72 h. Virus-infected wells were determined by the appearance of cytopathic effect (CPE) seen in the light microscope. Infection titer of viral stock was calculated by Reed and Muench method and expressed in log_10_TCID_50_/mL [10]. All procedures involved live SARS-CoV-2 were performed in the BSL-3 laboratory.

### 2.3. Assessment of Antibody Immune Responses

Humoral immunity to SARS-CoV-2 was assessed by ELISA and microneutralization assay (MN). ELISA was performed on the 96-well high-sorbent plates (Corning, Glendale, AZ, USA) using a recombinant protein corresponding to the receptor binding domain (RBD) of SARS-CoV-2 S-protein (BIOCAD, Saint Petersburg, Russia) as a coating antigen. The protein was coated at a concentration 100 ng per well in carbonate-bicarbonate buffer (pH 9.7) overnight at 4 °C. After blocking with 1% BSA in PBS (pH 7.4) for 1 h and washing with PBST (PBS with 0.05% of Tween 20), 2-fold sera dilutions were added to wells and incubated for another 1 h. After washing, goat anti-human IgG antibody conjugated to HRP (Sigma, St. Louis, MO, USA) was added at 1:10,000 dilution and incubated at 37 °C for 1 h. The plates were developed using 1-Step Ultra TMB-ELISA Substrate Solution (Thermo Fisher Scientific, Waltham, MA, USA). After 15 min incubation, the reaction was stopped with 1 M H_2_SO_4_ solution and the results were read at wavelength 450 nm using xMark Microplate Spectrophotometer (BioRad, Hercules, CA, USA). The endpoint antibody titer was determined as the last serum dilution with OD_450_ value exceeding two means of the optical density of control wells.

The 50% virus-neutralizing titers were determined as described in [9] with some modifications. Briefly, heat-inactivated serum samples were diluted 2-fold ranging from 1:10 to 1:640 dilutions using DMEM/2%FBS. Each serum sample was run in duplicates. The serum dilutions were mixed with 300 TCID_50_ of SARS-CoV-2 and incubated at 37 °C for 1 h. Then, the serum-virus mixture was transferred to Vero cells seeded on 96-well cell culture plates, followed by 1-h adsorption at 37 °C and 5%CO_2_. After this, the supernatants were carefully removed and the initial serum dilutions were added to corresponding wells in the cell culture plates. Finally, fresh DMEM/2%FBS was added to each well, and the plates were incubated at 37 °C and 5% CO_2_ for 48 h. After incubation, the plates were fixed with 10% formaldehyde at 4 °C for 24 h, for virus inactivation. The fixed plates were transferred to BSL-2 laboratory for cell-ELISA procedure. This included washing with PBS, cell permeabilization with 0.1% Triton X-100 at room temperature for 15 min, blocking with 3% skimmed milk at 37 °C for 1 h, followed by washing with PBS. Then, purified polyclonal rabbit anti-RBD antibody (BIOCAD, Saint Petersburg, Russia) was added at a concentration 1.5 µg/mL for 1 h, followed by washing with PBS and addition of secondary anti-rabbit IgG HRP-conjugated antibody for 1 h at 37 °C. The antibody binding was detected with TMB substrate as described above. To calculate the MN_50_ titer, four-parametrical non-linear regression analysis was used, based on the OD_450_ values of virus-negative and virus-positive cells as described in [9].

### 2.4. Assessment of Cellular Immune Responses

The T-cell immune response was assessed using an ICS assay with estimating of SARS-CoV-2-specific IFNγ levels. Fresh PBMCs were stimulated with live purified SARS-CoV-2 at different MOIs to select the optimal viral dose (1.0, 0.1, 0.01, 0.001 and 0.0001). Two million PBMC were seeded in 96-well plate in 100 µL of CR-10, followed by addition of 50 µL CR-10 containing the desired amount of purified SARS-CoV-2 or CR-10 without the virus for detection of nonspecific IFNγ production. After 18-h incubation at 37 °C and 5% CO_2_, 50 µL of CR-10 with Brefeldin A diluted 1:250 were added to each well. In addition, PMA and ionomycin mitogens were added to some wells as positive controls. Following 5-h incubation at 37 °C and 5%CO_2_, the cells were centrifuged at 500× *g*, 4 °C for 3 min, and the cells were stained with the following surface antibody cocktail at 4 °C for 20 min in the dark: CD3-PC7 (clone UCHT1, Biolegend, San Diego, CA, USA), CD4-APC-AF750 (clone 13B8.2, Beckman Coulter, Chaska, MN, USA), CD8-PC5.5 (clone B9.11, Beckman Coulter), CD45RA-ECD (clone 2H4, Beckman Coulter), CCR7-FITC (clone 150503, BD) antibodies, and ZombieAqua Staining for detection live/dead cells. Fixation and permeabilization were conducted using BD Cytofix/Cytoperm Fixation/Permeablization Kit (Becton Dickinson, Franklin Lakes, NJ, USA), according to the manufacturer’s instructions, followed by staining lymphocytes with an anti-IFNγ-PE (clone B27, BD) antibody for another 20 min. Then, the samples were washed twice with 200 μL of a wash buffer. The cells were fixed in 100 µL of Cyto-last buffer (Biolegend, San Diego, CA, USA) and stored in a dark cool place prior to the flow cytometric analysis. At least 500,000 events were measured using a Navios flow cytometer (Beckman Coulter, Brea, CA, USA). Single-stained samples were used for detector sensitivity calibration, as well as for compensation matrix calculation using autocompensation tools of flow cytometer; and the resulting compensation matrix was validated with the FMO controls. The data were analyzed using the FlowJo software (TriStar Inc., El Segundo, CA, USA); the proportion of virus-specific T cells was calculated by subtracting the negative control from the IFNγ-positive T cells. The gating strategy for the ICS assay is shown in Figure S1. For biological control of the assay, a subset of ten COVID-19-positive and five COVID-19-naïve PBMC specimens was stimulated with a sucrose-purified A/South Africa/3626/2013 (H1N1) influenza virus as previously described [11], with the exception that a different antibody cocktail for the ICS assay was used in the present study.

### 2.5. Statistical Analysis

Data were analyzed with the statistical module of Graph Pad Prism 6 software. Statistically significant differences between several study groups were determined by Kruskal−Wallis test with Dunn’s multiple comparisons test; the differences between two groups were determined by two-sided Mann−Whitney U test. *P* values of <0.05 were considered significant.

## 3. Results

**Figure 1:**
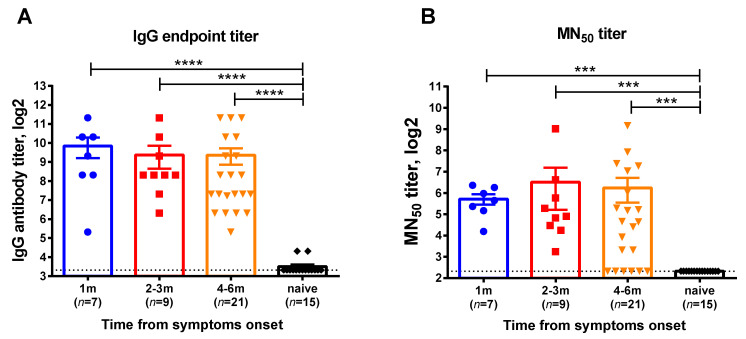
SARS-CoV-2 virus-specific antibody in serum samples of COVID-19 convalescents who participated in this study. (**A**) Serum IgG antibody titers determined in ELISA to RBD recombinant protein, (**B**) 50% virus-neutralizing titers determined by the MN_50_ assay. The bars indicate the mean values and the error bars show standard error mean values. Data were analyzed by Kruskal−Wallis test with Dunn’s multiple comparisons test. *** *p* < 0.001; **** *p* < 0.0001.

This cross-sectional cohort study involved participants who were diagnosed with COVID-19 by RT-PCR testing in Saint Petersburg, Russia, with the disease onset ranging from June 2020 till December 2020, when the Chinese SARS-CoV-2 strain prevailed in circulation. Serum samples and whole blood were collected at one or two time points for each subject corresponding to 1, 2–3, or 4–6 months after infection. The demographic characteristics of the blood donors divided into these test groups are shown in Table S1. Virus-specific IgG antibodies assessed by ELISA were detected in all participants regardless of the time elapsed after recovery (Figure 1A). In contrast, there were several subjects without neutralizing antibodies at the 6-month timepoint, however, the overall MN_50_ titers in all test groups were similar (Figure 1B). Although these data cannot be interpreted as the persistence of virus-specific antibodies over time, as different subjects were included in the different groups, all study participant can be considered as having specific immunity to SARS-CoV-2 and their eligibility for the assessment of virus-specific memory T-cell responses.

To assess virus-specific memory T-cell responses, we developed a protocol for stimulating the PBMCs of the donors who had recovered from COVID-19 with live SARS-CoV-2. The virus was successfully purified on a 30/60% sucrose gradient, with a resulting infectious titer 10^7.5^ TCID_50_/mL. To find an optimal viral dose for the ICS assay, the PBMCs of a COVID-19 convalescent collected 1 month after disease onset were stimulated with the virus at MOIs 1.0, 0.1, 0.01, 0.001 and 0.0001. For positive control, PMA/ionomycin mixture was used for stimulation. Strikingly, the IFNγ-producing CD4 and CD8 effector memory T cells were detected at significantly higher levels when relatively low doses of the virus were used for stimulation (MOI 0.01 and 0.001, Figure S2).

**Figure 2:**
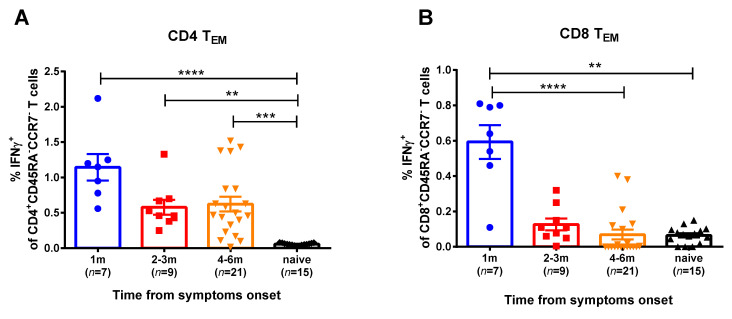
SARS-CoV-2 virus-specific CD4^+^ (**A**) and CD8^+^ (**B**) memory T cells in subjects recovered from COVID-19. Proportions of IFNγ-secreting cells among corresponding Tem cell subsets were measured by ICS assay using PBMCs collected at various time after the disease onset. The bars indicate the mean values and the error bars show standard error mean values. Data were analyzed by Kruskal−Wallis test with Dunn’s multiple comparisons test. ** *p* < 0.01; *** *p* < 0.001; **** *p* < 0.0001.

For the current study, SARS-CoV-2 at an MOI 0.001 was used for in vitro stimulation of the PBMC samples of the COVID-19 convalescents. We studied two memory subsets that mediate recall responses to a pathogen: central memory (CD45RA^-^CCR7^+^) and effector memory (CD45RA^-^CCR7^-^) T helpers (CD4^+^) and cytotoxic T lymphocytes (CD8^+^). Naïve T cells were also assessed, however, they did not produce IFNγ, as expected (data not shown). The T-helper effector memory cells of convalescents produced higher IFNγ levels in comparison with naïve group, up to 6 months after SARS-CoV-2 infection (Figure 2, Figure S3). In contrast, the SARS-CoV-2 virus-specific CTLs were detected in the peripheral blood only for a month after infection, whereas at 2 months and later this subset was not significantly different between convalescents and naïve subjects. Central memory T cells did not respond to viral stimulation (data not shown). Importantly, high proportions of IFNγ-secreting CD4^+^ and CD8^+^ T cells were observed when the PBMCs were stimulated with human influenza A virus, regardless of the COVID-19 infection status and the time that had elapsed from symptom onset (Figure S4). Since influenza virus epidemics have not been observed in the study location for more than a year, these data clearly indicate that both cytotoxic and helper memory T-cell responses to influenza virus antigens persist in circulation for a long time after infection, whereas SARS-CoV-2-reactive CD8^+^ Tem subset rapidly decline after infection.

## 4. Discussion

It is known that serum IgG antibody levels, as well as neutralizing antibody titers decline over time in COVID-19 recovering patients, as was established in well-controlled longitudinal observational studies [12,13]. Therefore, it is especially important to understand for how long the virus-specific T cells are maintained, as they can rapidly respond to re-infection and protect individuals by activating robust antibody production and or via the cytotoxicity mechanism leading to fast elimination of the pathogen from the infected organism [14]. The persistence of T-cell responses after SARS-CoV-2 infection was observed for up to 8 months after infection using peptide pools covering all proteins of SARS-CoV-2, however, the authors suggested that the 15-mer peptides can be suboptimal for the detection of some antigen-specific T-cell subsets [15]. The use of predicted SARS-CoV-2 class I epitopes of optimal size can also underestimate the level of virus-specific CD8^+^ T cells in COVID-19 convalescents due to the limitations in epitope-predicting algorithms, especially for CD4^+^ T cells [16]. Even stimulation with inactivated whole SARS-CoV-2 [8] cannot be translated to the in vivo situation due to the nonstructural proteins that are not present within a virion but are expressed inside infected cells; and the T cells specific to the epitopes within such proteins will not be captured unless the live virus is used in the assay. Therefore, the use of live SARS-CoV-2 for in vitro stimulation of immune cells seems more biologically relevant than stimulation with peptides or peptide pools due to the unique antigen-presenting pathways for every subject, owing to the diversity of MHC alleles in the population. In this case, the autologous antigen-presenting cells are involved in antigen processing and epitope presentation to the T cells, thus representing the maximal repertoire of virus-specific T cells for each particular individual. Here, we report for the first time the preliminary data of SARS-CoV-2 live virus-specific T-cell responses in convalescent and naïve to COVID-19 donors up to 6 months after the disease onset, while the study of the longevity of these responses is underway.

Unlike the influenza virus, the optimal SARS-CoV-2 viral dose for PBMC stimulation was fairly low (3–5 MOI for influenza versus 0.001 MOI for coronavirus) [11]. These differences may arise from the different biological pathways these viruses use to infect the susceptible cells. Influenza virus and SARS-CoV-2 have different receptor specificities and are characterized by different immunological milieu at the site of infection [17]. Furthermore, the influenza virus requires proteolytic cleavage of the hemagglutinin molecule for successful multicycle replication [18], whereas SARS-CoV-2 produces up to 10^3^ virions every 10 h with a potential to infect cells which express ACE2 receptor [19]. As a result, the higher SARS-CoV-2 doses can have deleterious effects on the expression of surface markers on the immune cells, thus resulting in the decrease of the total CD4^+^/CD8^+^ populations in the virus-stimulated specimens. In addition, it was previously demonstrated that MERS-CoV, but not SARS-CoV, efficiently infected T cells and induced substantial apoptosis in the infected cells [20]. Furthermore, the S1 protein of another coronavirus—porcine epidemic diarrhea virus—could induce cell apoptosis, suggesting that the spike protein can be an effective inducer of cell apoptosis in vivo [21].

As shown in the current study, the low doses of SARS-CoV-2 successfully stimulated virus-specific Tem populations in the circulating blood. The persistence of CD4^+^ Tem cells in circulation over time after COVID-19 recovery indicates that the virus-specific antibody levels can be maintained, conferring protection against re-infection [22]. Of note, T follicular helper (Tfh) cells—a subset of CD4^+^ T cells—are essential for the generation of plasma and memory B cells during the germinal center reaction, being equipped with the different features required for effective B cell help [23]. Nowadays, the data about SARS-CoV-2-specific Tfh cells are very limited, but our findings indirectly indicate the presence of this Th cell subset within the total pool of memory T cells.

Furthermore, the detected decline in the levels of SARS-CoV-2 virus-specific CD8^+^ Tem in peripheral circulation can be either due to the disappearance of these memory T cells from the organism or due to their migration from lymphoid to peripheral tissues and becoming T_RM_ cells, which are important for future protection against SARS-CoV-2 infection [24]. In the study by Dan et al. SARS-CoV-2-specific CD4^+^ T cells and CD8^+^ T cells declined over time with a half-life 3 to 5 months, and the majority of SARS-CoV-2-specific memory CD8^+^ T cells were terminally differentiated effector memory cells (CD45RA^+^CCR7^-^), with small populations of central memory (CD45RA^-^CCR7^+^) and effector memory (CD45RA^-^CCR7^-^), while a plurality of the SARS-CoV-2 memory CD4^+^ T cells present at ≥ 6 months after infection had a CD45RA^-^CCR7^+^ central memory phenotype [15]. Next, Neidleman et al. also showed that the majority of SARS-CoV-2-specific CD8^+^ T cells were predominantly TEMRA cells in a state of less terminal differentiation than most TEMRA cells [25].

A limitation of this study is that the PBMC samples were collected from different individuals at different time points, and therefore it was not possible to assess individual kinetics of Tem responses for each subject. Nevertheless, the trend for the reduction of virus-specific CD8^+^, but not CD4^+^, Tem cells in circulation was clearly identified in this study. Another limitation is that we did not compare the results of the PBMCs’ whole virus stimulation with contemporary methods of T-cell analysis using peptide pools or some viral proteins for in vitro stimulation of the immune cells. As previously identified, the M, S, and N proteins were codominant and could be recognized by 100% of COVID-19 cases [7]. In addition, significant CD4^+^ T-cell responses were directed against nsp3, nsp4, ORF3s, ORF7a, nsp12, and ORF8. In the cases of SARS-CoV-2-specific CD8^+^ T cells, the S and M proteins were targets of human responses; however, significant reactivity was noted for other antigens, including nsp6, ORF3a, and N, which activated, on average, almost 50% of the total CD8^+^ T-cell responses [7].

As for memory phenotype, the majority of SARS-CoV-2-specific CD4^+^ T cells in COVID-19 convalescents were identified as central memory T cells when stimulated with MegaPools (overlapping or prediction-based peptides covering the SARS-CoV-2 proteome) [26]. In contrast, the phenotyping of activated CD8^+^ T cells showed that the majority of virus-specific CD8^+^ T cells were of CD45RA^–^CCR7^–^ effector memory or terminally differentiated effector (CD45RA^+^CCR7^–^) phenotypes. Furthermore, staining the PBMCs of the COVID-19 convalescents with MHC-I multimers revealed that SARS-CoV-2-specific CD8^+^ T cells had effector memory (CD45RA^−^CCR7^−^) or central memory (CD45RA^−^CCR7^+^) phenotypes with early (CD27^+^CD28^+^) or intermediate (CD27^+^CD28^−^) differentiation [27]. Overall, more research is needed to establish the correlation between antigen-specific memory T-cell responses identified by stimulating PBMCs with live SARS-CoV-2 and particular peptide pools.

## 5. Conclusions

The developed assay of stimulating human PBMC specimens with live SARS-CoV-2 can successfully detect virus-specific populations of CD4^+^ and CD8^+^ memory T cells in patients who have recovered from COVID-19. This method is most suitable for assessing the levels of virus-specific T cells induced by SARS-CoV-2 T cell-based vaccine candidates under development. Unlike conventional T cell-based assays used for the assessment of cellular immune responses in infected or vaccinated people, the presented methodology can estimate the vaccines’ ability to establish long-lived memory T-cell responses to the whole virus, which can be considered as a possible immune correlate of protection. Although this study was conducted with a SARS-CoV-2 strain isolated during the first COVID-19 wave, future studies will identify whether the virus-specific T cells established by natural infection can cross-react with recently emerged SARS-CoV-2 variants, providing the important mechanism of immune defense against reinfection with antigenically evolved viruses.

## Data Availability

The data presented in this study are available on request from the corresponding author.

## Supplementary Materials

The following are available online at https://www.mdpi.com/article/10.3390/v13081490/s1. Figure S1: Gating strategy for the IFNγ ICS assay; Figure S2: IFNγ production by CD4^+^ memory T cells after stimulation with various doses of live SARS-CoV-2; Figure S2: Representative gates for IFNγ production by CD8^+^ and CD4^+^ memory T cells; Figure S3: Representative gates for IFNγ production by CD8^+^ (upper panel) and CD4^+^ (lower panel) memory T cells after in vitro stimulation of PBMCs isolated from COVID-19 convalescent (left panel) or a naïve subject (right panel) with live SARS-CoV-2 virus; Figure S4: Influenza virus-specific CD4^+^ (A) and CD8^+^ (B) memory T cells in subjects recovered from SARS-CoV-2 infection and in COVID-19-naïve participants. Table S1: Demographic characteristics of the study subjects.
