# Supplementary material for: Detection of IFNγ-Secreting CD4+ and CD8+ Memory T Cells in COVID-19 Convalescents after Stimulation of Peripheral Blood Mononuclear Cells with Live SARS-CoV-2
Source: Viruses. 2021 Jul 29;13(8):1490. doi: 10.3390/v13081490 (PMC8402842; doi:10.3390/v13081490)
Supplement: Supplementary file 1 [file viruses-13-01490-s001.zip › viruses-1301097-supplementary.pdf]

Supplementary materials

Table S1. Demographic characteristics of the study subjects.

Group	Time from Disease Onset, Months	*n*	Age, Years Mean (Range)	Sex		Chronic Diseases, *n* (%)	Disease Severity	
				Female, *n* (%)	Male, *n* (%)		Mild, *n* (%)	Moderate to Severe, *n* (%)
1	1	7	42 (24–61)	5 (71.4)	2 (28.6)	0 (0.0)	7 (100)	0 (0)
2	2-3	9	43.9 (32–77)	6 (66.7)	3 (33.3)	1 (11.1) †	6 (66.7)	3 (33.3)
3	4-6	21	40.5 (25–61)	13 (61.9)	8 (38.1)	2 (9.5) #	15 (71.4)	6 (28.6)
Naïve	n/a	15	35.9 (21-68)	9 (60.0)	6 (40.0)	1 (6.7) ‡	n/a	n/a

† atopic dermatitis; # chronic bronchitis and Hashimoto's thyroiditis; ‡ hypertonic disease; n/a: not applicable.

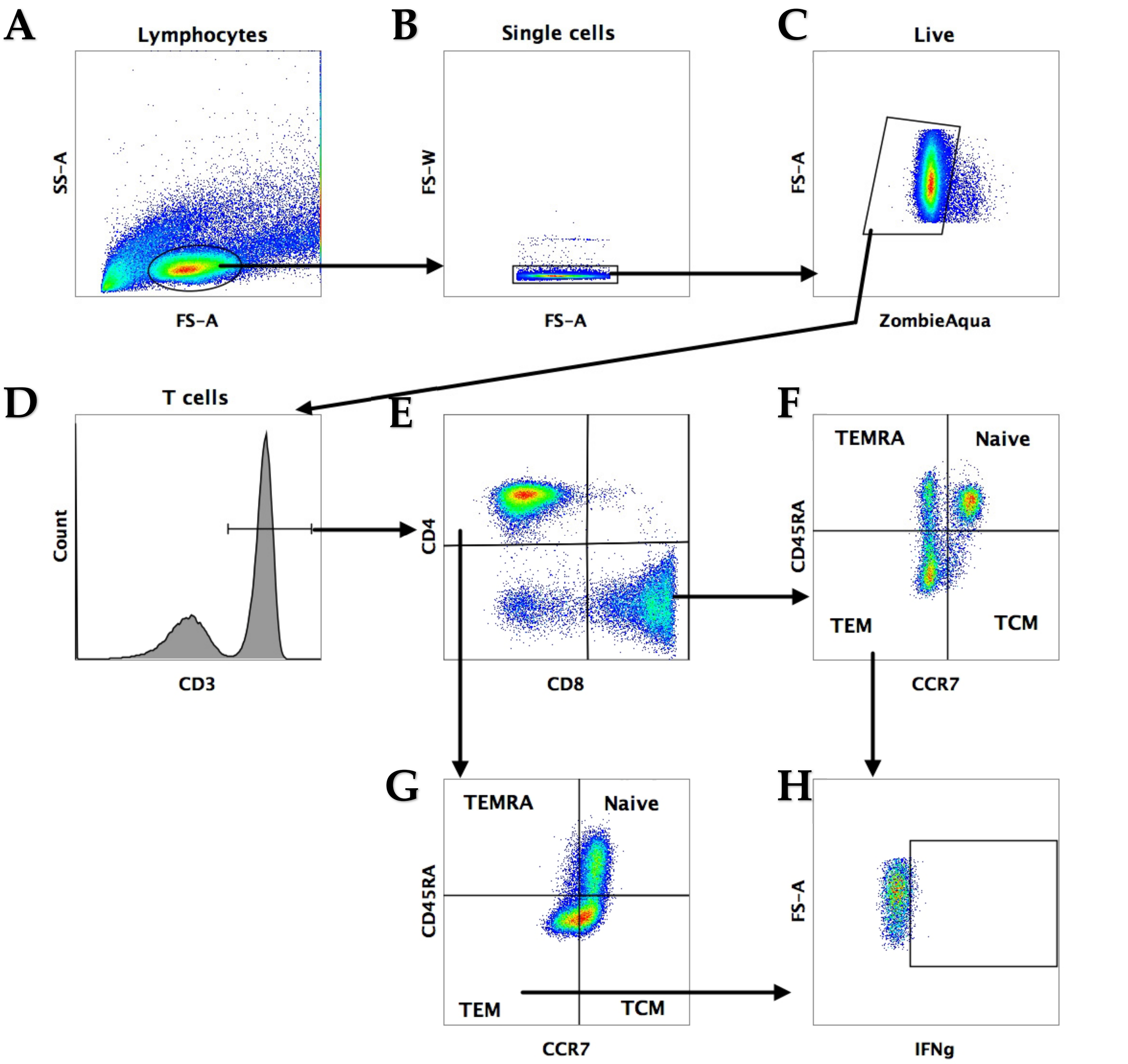

Figure S1. Gating strategy for the IFNγ ICS assay. Total lymphocytes were gated based on their side-scatter and forward-scatter (dot plot **A**); dot plot **B** – doublets were excluded from the analysis using FS-area (FS-A) and FS-height (FS-W) and ZombieAqua-positive dead cells were eliminated from lymphocytes (dot plot **C**). Next, based on $CD3^+$ expression (dot plot **D**) the total T cell subset was identified. T cells were further analyzed by expression of $CD8^+$ and $CD4^+$ (dot plot **E**) for identification of total $CD3^+CD8^+$ cytotoxic T cells and $CD3^+CD4^+$ T helper cells. Dot plots **F** and **G** - within total $CD3^+CD8^+$ and $CD3^+CD4^+$ cells we purified "naïve" ($CD45RA^+CCR7^+$), central memory ($CD45RA^-CCR7^+$), effector memory ($CD45RA^-CCR7^-$) cells and TEMRA ($CD45RA^+CCR7^-$) cells. Finally, within $CD45RA^-CCR7^-$ TEM cytotoxic T cells and Th cells IFNγ-expressing cells were identified (dot plot **H** shown as an example).

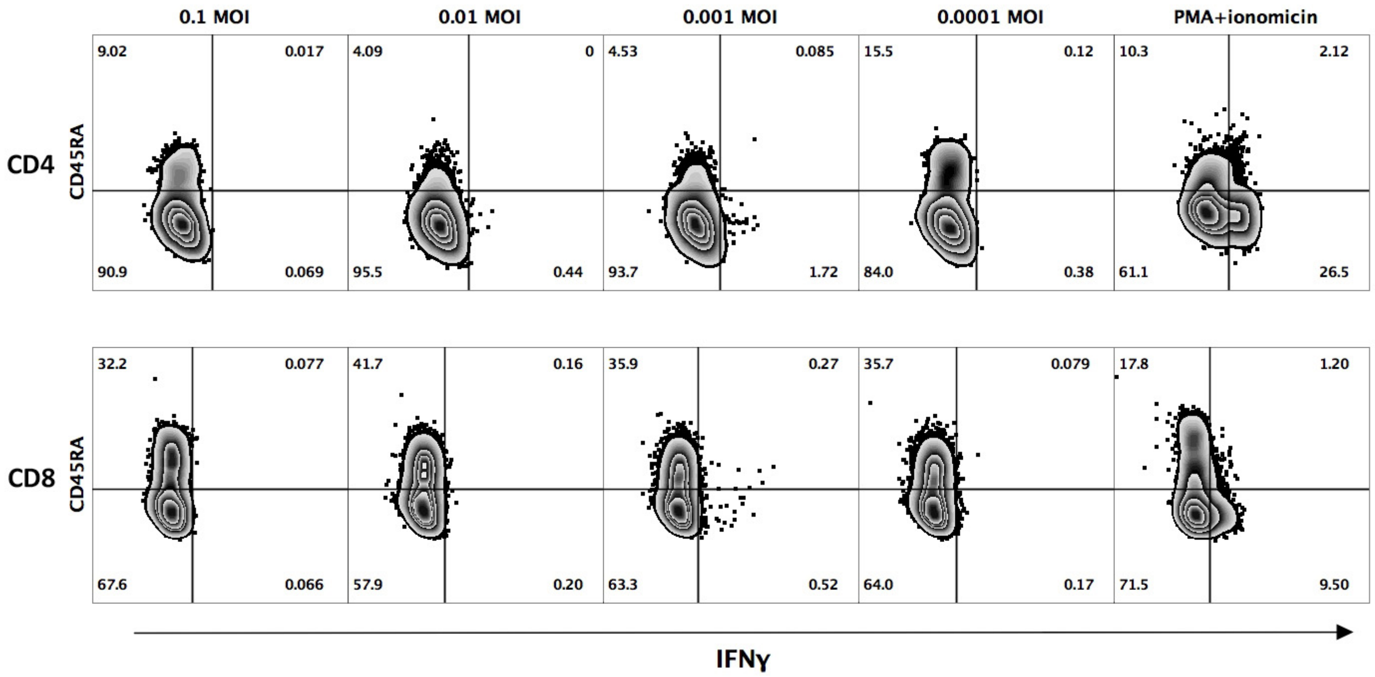

Figure S2. IFNγ production by $CD4^+$ (upper panel) and $CD8^+$ (lower panel) memory T cells after stimulation with various doses of live SARS-CoV-2. Fresh PBMCs isolated from COVID-19 recovered patient were stimulated in vitro with different concentrations of SARS-CoV-2. IFNγ production by $CD4^+$ and $CD8^+$ T cells was assessed by flow cytometry.

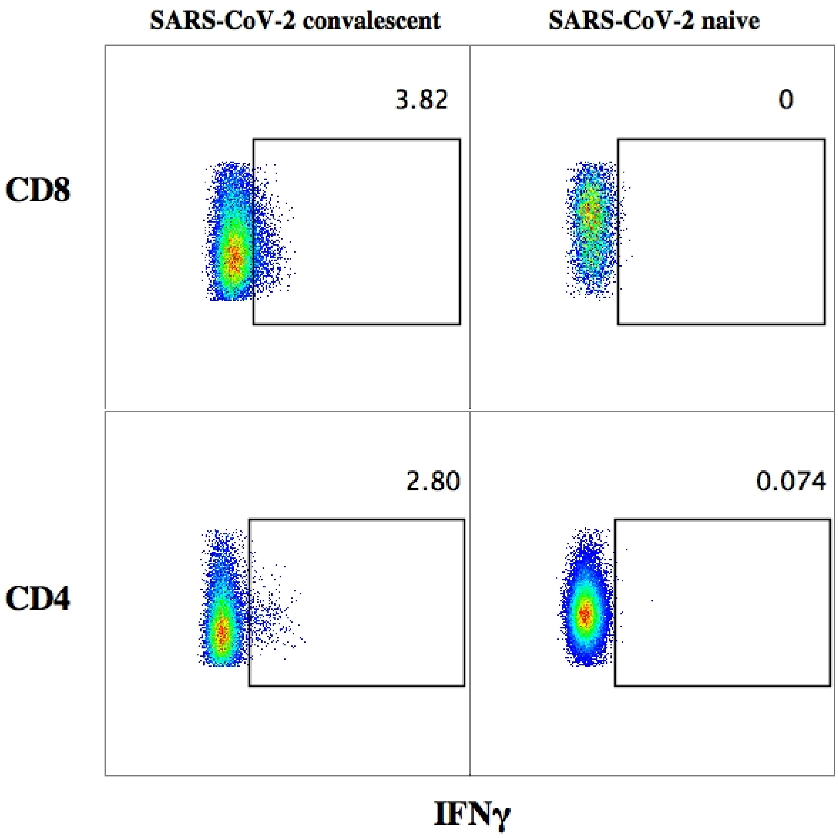

Figure S3. Representative gates for IFNγ production by CD8⁺ (upper panel) and CD4⁺ (lower panel) memory T cells after in vitro stimulation of PBMCs isolated from a COVID-19-recovered subject one month after the disease onset (left panel) or a naïve subject (right panel) with live SARS-CoV-2.

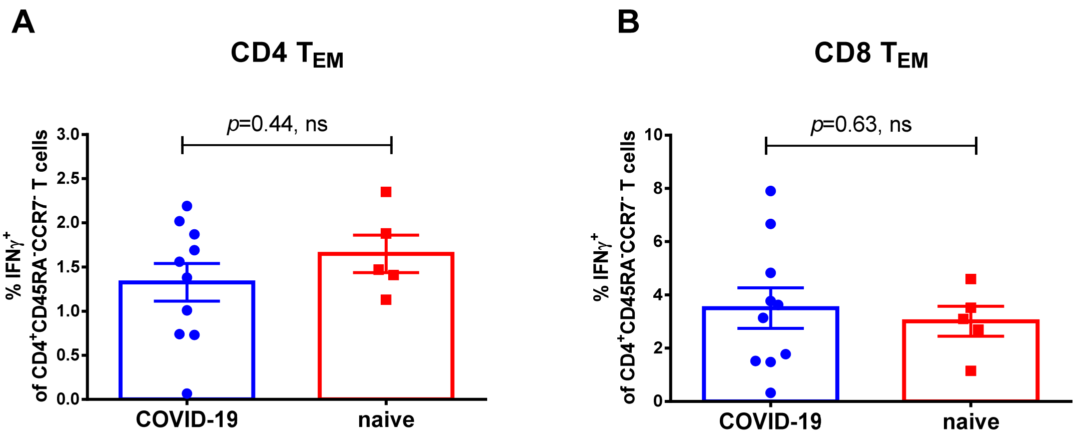

Figure S4. Influenza virus-specific CD4⁺ (**A**) and CD8⁺ (**B**) memory T cells in subjects recovered from SARS-CoV-2 infection and in COVID-19-naïve participants. Proportions of IFNγ-secreting cells among corresponding Tem cell subsets were measured by ICS assay. The bars indicate the mean values and the error bars show standard error mean values. Data were analyzed by two-sided Mann-Whitney U test. ns: not significant.
